# OCT angiography characterizes time- and diameter-dependent intestinal microvascular alterations in acute mesenteric ischemia

**DOI:** 10.1117/1.JBO.31.11.116001

**Published:** 2026-11-09

**Authors:** Yu Tian, Feifan Wang, Jian Liu, Nan Li, Mingshuo Zhang, Rui Ding, Yimin Wang

**Affiliations:** a First Hospital of Qinhuangdao, Department of Hand & Foot Surgery; Qinhuangdao, China; b First Hospital of Qinhuangdao, Department of General Surgery, Qinhuangdao, China; c Northeastern University at Qinhuangdao, School of Control Engineering, Qinhuangdao, China; d Qinhuangdao Maternal and Child Health Hospital, Department of Ophthalmology, Qinhuangdao, China; e Hebei North University, Zhangjiakou, China; f Hebei Medical University, Shijiazhuang, China

**Keywords:** optical coherence tomography, acute mesenteric ischemia, microvascular dysfunction, intestinal microcirculation, vessel perfused density, intestinal mucosal injury

## Abstract

**Significance:**

Acute mesenteric ischemia (AMI) is associated with high mortality, and accurate assessment of intestinal viability and the extent of ischemic injury remains challenging. Microvascular alterations may precede overt mucosal structural damage but are difficult to monitor dynamically.

**Aim:**

We aim to investigate the ability of OCT/OCTA to dynamically quantify intestinal mucosal microvascular alterations during AMI and to determine their temporal and vessel-diameter-dependent associations with histological injury.

**Approach:**

AMI was induced in mice by mesenteric artery ligation. In a longitudinal cohort (n=8), OCT was performed at baseline and 0.5, 1, 2, and 4 h after ischemia to quantify vessel length and vessel perfused density (VPD) in three diameter categories (<20  μm, 20 to 50  μm, and >50  μm). In a separate cross-sectional cohort (n=40), mice were randomly assigned to sham or ischemia groups (n=8 each). OCT was also used to detect the vascular parameters of intestinal mucosal microcirculation. Histological injury was assessed by villus height, crypt depth, and Chiu’s score. Correlations between microvascular and histological parameters were analyzed. Receiver operating characteristic curve was used to assess vascular parameters for discriminating severe intestinal injury.

**Results:**

Ischemia induced time- and diameter-dependent microvascular impairment. With the prolongation of ischemia, both vessel length and VPD decreased significantly across all vessel diameter categories (P<0.05). The length and VPD of vessels >50  μm decreased significantly faster than those of vessels <20  μm (P<0.05). Notably, reductions in the vascular parameters of 20 to 50  μm and >50  μm vessels were observed as early as 0.5 h after ischemia and preceded significant elevation of Chiu’s score. Villus height and crypt depth progressively decreased, whereas Chiu’s score increased with prolonged ischemia. OCT-derived vascular parameters across all diameter categories were positively correlated with villus height and crypt depth and negatively correlated with Chiu’s score (all P<0.05). In addition, OCT-derived vascular parameters had significant diagnostic performance for severe mucosal injury (${\mathrm{Chiu}}^{\prime }s\;\mathrm{score}\geq 3$).

**Conclusions:**

OCT enables real-time and quantitative assessment of intestinal mucosal microvascular alterations in AMI. Diameter-dependent microvascular impairment precedes overt histological injury and is associated with the severity of mucosal damage. These findings support the potential use of OCTA for intraoperative assessment of intestinal viability and provide a basis for future identification of ischemic transition zones and optimization of bowel resection boundaries.

## 1 Introduction

Acute mesenteric ischemia (AMI) is a life-threatening abdominal emergency characterized by sudden interruption of mesenteric blood flow, leading to rapid deterioration of intestinal viability and high mortality rates if not diagnosed and treated promptly.^1^^,^^2^ Despite advances in surgical and critical care management, the prognosis of AMI remains poor, largely due to difficulties in early diagnosis and timely assessment of intestinal injury severity.^1^ Early ischemic injury primarily involves microcirculatory dysfunction, which precedes irreversible mucosal damage.^3^^,^^4^ However, this stage is often clinically silent and difficult to detect using conventional diagnostic methods.

The intestinal mucosa is highly sensitive to ischemia because of its high metabolic demand and unique microvascular architecture. The mucosal microcirculation is organized as a hierarchical vascular network, consisting of arteriolar branches supplying villous arterial trunks, capillary networks within villi and crypts, and venous drainage systems.^5^ Disruption of this finely regulated microcirculatory system leads to impaired oxygen and nutrient delivery, resulting in villous edema, epithelial shedding, and crypt destruction.^3^^,^^6^ Histopathological assessment, such as the Chiu’s score, is widely regarded as the gold standard for evaluating intestinal mucosal ischemic injury.^7^ However, it primarily reflects established morphological damage and cannot capture early, potentially reversible microcirculatory alterations.

Current clinical imaging modalities for AMI, including computed tomography angiography and ultrasonography, are mainly effective in identifying macroscopic vascular occlusion and secondary intestinal changes, such as bowel dilation or wall thickening.^2^^,^^8^ These techniques have limited sensitivity for detecting early microcirculatory dysfunction. Invasive approaches, such as mesenteric angiography, although capable of visualizing vascular patency, carry procedural risks and are not suitable for repeated or dynamic monitoring in the operation.^9^ Therefore, there is an unmet need for an objective imaging approach that can provide real-time and quantitative information on intestinal microcirculation during surgical exploration. In patients undergoing surgery for AMI, macrovascular occlusion and ischemia may already have been established by the time of intervention. A major clinical challenge is then to determine the viability of ischemic intestinal segments and to distinguish severely injured bowel from potentially salvageable tissue. This challenge is particularly relevant in bowel segments with an equivocal appearance, in which conventional assessment based on color, peristalsis, mesenteric pulsation, and bleeding remains subjective. An imaging modality capable of quantitatively characterizing local intestinal perfusion may therefore provide complementary information for intraoperative assessment of bowel viability.

Optical coherence tomography (OCT) is a high-resolution, label-free optical imaging modality that provides cross-sectional and angiographic visualization of tissue microstructure and microvasculature *in vivo*.^10^ OCT angiography enables quantitative evaluation of microvascular parameters, such as vessel length and perfused vessel density, without the need for exogenous contrast agents.^11^ Owing to its micrometer-scale resolution and real-time imaging capability, OCT has been widely applied in ophthalmology and increasingly explored for microcirculatory assessment in other organs.^12^^,^^13^ Previous clinical studies have suggested that multimodal OCT may provide valuable information for intraoperative assessment of intestinal viability and identification of bowel resection boundaries in patients with AMI.^14^ However, the potential value of OCT for dynamic monitoring of intestinal mucosal microcirculation during AMI, particularly with respect to vessel diameter–dependent alterations and their relationship with histological injury, remains insufficiently investigated.

Our previous study investigated the progression of acute intestinal ischemia using swept-source optical coherence tomography angiography and demonstrated differential changes in vascular perfusion density, vessel length, and average vessel diameter across different intestinal wall layers.^15^ Although these findings established the feasibility of OCTA for monitoring ischemia-associated microvascular alterations, the influence of vessel caliber within the intestinal mucosal microvascular network remained unclear. In particular, whether different vessel diameters exhibit distinct temporal responses to ischemia and whether these alterations are associated with the severity of histological mucosal injury have not been determined. The present study was therefore designed to extend our previous work by focusing specifically on diameter-dependent microvascular alterations within the intestinal mucosa and by linking these OCTA-derived changes to histological injury.

## 2 Materials and Methods

### 2.1 Animal Model of Acute Mesenteric Ischemia

All animal experiments were approved by the Ethics Committee of the First Hospital of Qinhuangdao (No. 2022k016). A total of 48 male C57BL/6 mice (8 to 10 weeks old, weighing 22 to 25 g) were used in this study and housed under standard laboratory conditions with free access to food and water. Acute mesenteric ischemia was induced by superior mesenteric artery (SMA) ligation as previously described, with minor modifications.^16^ Briefly, mice were anesthetized using isoflurane gas and placed in a supine position on a temperature-controlled heating pad during the experiment. After midline laparotomy, the SMA was carefully exposed and permanently ligated using a 6 to 0 silk suture to establish an acute occlusive ischemia model. Successful occlusion was confirmed by loss of mesenteric arterial pulsation. Sham-operated mice underwent identical surgical procedures without SMA ligation.

### 2.2 Experimental Design

Figure 1 illustrates the overall study design. This study consisted of two parts. In the first part, an acute mesenteric ischemia mouse model was established (n=8). The same animals were followed longitudinally throughout the experiment. OCT/OCTA imaging was performed in the same mouse at baseline (before SMA ligation) and subsequently at 0.5, 1, 2, and 4 h after SMA ligation. Therefore, repeated measurements at all predefined time points were obtained from the same animals, with each mouse serving as its own baseline reference. Following SMA ligation, the animals remained under continuous anesthesia throughout the imaging period. Anesthesia was induced with 3% to 4% isoflurane and maintained with 1% to 2% isoflurane delivered in oxygen throughout the imaging period. Body temperature was maintained at ∼37°C using a thermostatically controlled heating pad throughout the imaging period. Respiratory status and general physiological condition were monitored throughout the experiment. At each predefined time point, OCT/OCTA imaging was performed under the maintained anesthetic and physiological conditions using the same intestinal segment.

**Fig. 1:**
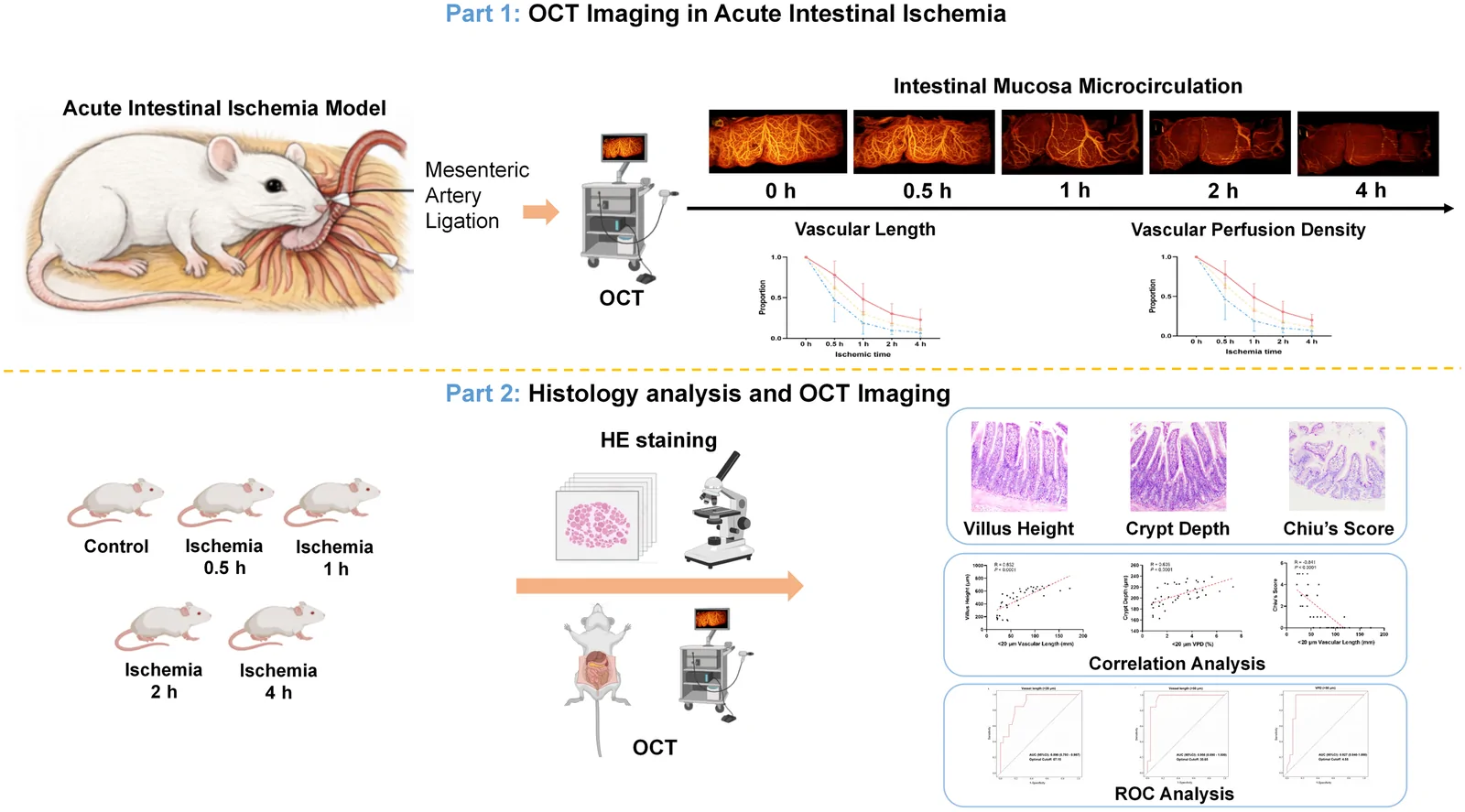
Flowchart of the study design.

In the second part, 40 mice were randomly assigned to five groups (n=8 per group): (1) sham-operated control group, (2) 0.5-h ischemia group, (3) 1-h ischemia group, (4) 2-h ischemia group, and (5) 4-h ischemia group. At the end of the assigned ischemic period, the occlusion was released to restore mesenteric blood flow. After reperfusion, OCT/OCTA imaging was then performed immediately to evaluate intestinal mucosal microvascular parameters in each group. Subsequently, hematoxylin and eosin (H&E) staining was used to assess intestinal mucosal histological parameters, including villus height, crypt depth, and Chiu’s score. For both OCT imaging and histopathological analysis, an intestinal segment located 5 cm proximal to the ileocecal junction was selected.

### 2.3 Histological Assessment of Intestinal Injury

Following OCTA imaging at each predefined ischemic time point, the mice were immediately euthanized. The imaged intestinal segment was then rapidly harvested, and tissue fixation was initiated within ∼5  min after completion of OCTA acquisition. Tissue samples were fixed in 4% paraformaldehyde, embedded in paraffin, sectioned at 4  μm, and stained with hematoxylin and eosin (H&E). Intestinal mucosal injury was evaluated using the Chiu’s scoring system, ranging from grade 0 (normal mucosal architecture) to grade 5 (severe mucosal destruction with hemorrhage and ulceration).^7^ Villus height and crypt depth were measured using ImageJ software (NIH, United States) from well-oriented sections. At least five villi and crypts per section were analyzed, and mean values were calculated for each mouse. Histological assessments were performed by two independent pathologists blinded to the experimental conditions.

### 2.4 Optical Coherence Tomography Angiography

The OCTA system operates at a central wavelength of 1310 nm with a spectral bandwidth of 100 nm, and the sample arm power is 3 mW.^17^^,^^18^ The system achieves an axial resolution of ∼8  μm and a lateral resolution of 15  μm, with a penetration depth of about 2 mm in biological tissue. The line scan rate is 200 kHz. The OCTA device is equipped with a flexible fiber probe, at the distal end of which is a detachable filter with an outer diameter of 20 mm. Imaging of intestinal tissue was performed using a contact method. Each B-scan consists of 1000 A-scans, and five repeated scans were acquired consecutively at each position; the frame with the lowest average correlation with the remaining four frames was discarded, and the remaining four scans were used for angiography. A total of 500 B-scans were collected along the slow axis, and the acquisition of a complete three-dimensional dataset took ∼15.6  s. A decorrelation algorithm was applied to suppress static tissue signals while retaining dynamic tissue signals originating from blood flow (i.e., blood flow information). The decorrelation value at each pixel was calculated from adjacent amplitude frames B_n(x,z) and B_n+1(x,z) as

$$\overset{¯}{D}(x,\;z)\;=\;1\;-\;\frac{1}{N\;-\;1}\;\cdot \;\overset{N\;-\;1}{\underset{n\;=\;1}{\sum }}\frac{B_{n}(x,\;z)\cdot B_{n\;+\;1}(x,\;z)}{1/2{B_{n}}^{2}(x,\;z)\;+\;1/2{B_{n\;+\;1}}^{2}(x,\;z)},$$ (1)

where B_n is the OCT amplitude of the *n*’th retained frame and N=4 is the number of retained repeated frames.^19^ Static tissue yields D close to 0, whereas flowing blood yields values approaching 1. The inter-frame interval was 6.25 ms (200 kHz line rate, 1000 A-lines per frame, 80% duty cycle). *En face* angiograms were obtained by averaging the decorrelation values along depth over ∼2  mm (about 230 pixels, 8.5 um per pixel) from the sample surface, covering a field of view of 10  mm(fast axis)×5  mm(slow axis).

### 2.5 Motion Management during *In Vivo* Imaging

Because imaging was performed in live animals, bulk motion and physiological motion (respiration, heartbeat, and intestinal peristalsis) were addressed at three levels. First, motion was reduced physically: imaging was performed under continuous isoflurane anesthesia with the animal placed on a thermostatically controlled heating pad, and the detachable filter at the distal end of the probe was brought into gentle contact with the intestinal surface, which mechanically stabilized the imaged segment and suppressed the majority of bulk motion before any signal processing. Second, at the acquisition level, five frames were acquired consecutively at each lateral position, and the frame showing the lowest average correlation with the remaining four frames was discarded before the decorrelation calculation, thereby removing frames corrupted by transient bulk motion. Third, after *en face* projection and before vessel binarization, the angiograms were processed with a 3×3 median filter to suppress residual motion-induced noise, including striping and isolated outliers produced by respiration and heartbeat. Image-based registration was deliberately not applied: contact imaging had already suppressed most bulk motion, frame-by-frame registration would have been computationally expensive relative to the expected benefit, and aggressive registration tends to attenuate the very decorrelation signal that carries the flow information. Together, these three measures yielded angiograms of high quality without motion correction by registration.

### 2.6 Quantification of Vascular Length and Vascular Average Diameter

To investigate changes in the length of vessels with different diameters during the progression of intestinal ischemia, the filtered OCTA intestinal angiogram was first binarized. In this study, the local adaptive region growing algorithm was used to extract vessel pixels.^20^ This algorithm automatically selects initial seed points based on the grayscale histogram, then dynamically updates thresholds within local regions, and continuously merges neighboring pixels that meet similarity criteria into the vessel region until no further growth is possible. The algorithm adapts its growth criteria to different regions, making it suitable for OCTA images with significant variations in vessel pixel intensity.

Next, vessel skeletons were extracted from the binarized vessel images using a morphology-based thinning algorithm. This algorithm iteratively removes boundary pixels while preserving connectivity, gradually reducing vessel structures to a single-pixel-wide skeleton. To incorporate vessel diameter information into the skeleton map, a distance transform was applied to the binarized vessel image. Each pixel value after distance transform represents the shortest distance from that point to the vessel boundary (i.e., the radius), and the diameter was obtained by multiplying by two. The distance-transformed image was then multiplied pixel-wise with the vessel skeleton image, yielding a vessel skeleton map encoding diameter information. In this map, each pixel value represents the vessel diameter at that location, and the vessel length is calculated as the number of skeleton pixels multiplied by the pixel size.

Vessel quantification included calculating the total length of perfused vessels (LL, in mm), which was performed using a Python 3.6 program. In addition, intestinal mucosal microvessels were categorized into three diameter groups (<20  μm, 20 to 50  μm, and >50  μm) to reflect the hierarchical organization of the microcirculation. Vessels <20  μm primarily correspond to capillary networks, whereas vessels between 20 to 50  μm and >50  μm represent arteriolar and upstream feeding vessels, respectively, which play distinct roles in oxygen delivery and flow regulation.^3^^,^^21^^,^^22^ The automated post-processing workflow consisted of binarization, skeletonization to extract the centerline of each vessel, summation of all skeleton pixels, and conversion of the resulting value to millimeters.

Vessel perfused density (VPD) was defined as the ratio of the number of vessel pixels to the total number of pixels in the *en face* angiogram.^23^ For the diameter-stratified analysis, VPD was calculated separately for each diameter category as the number of binarized vessel pixels belonging to vessels within that category divided by the total number of pixels of the *en face* angiogram, and is expressed as a percentage. Binarization by the locally adaptive region growing algorithm described above was the only segmentation step applied before this calculation.

### 2.7 Statistical Analysis

Continuous variables were presented as mean ± standard deviation or the interquartile range containing the median. Statistical analyses were performed using SPSS software version 22.0 and GraphPad Prism 7.0 software. For OCT-derived microvascular parameters measured repeatedly at different ischemic time points within the same animals, repeated-measures analysis of variance (repeated-measures ANOVA) was used to evaluate time-dependent changes followed by Tukey’s post hoc test. For comparisons of histological parameters (villus height, crypt depth, and Chiu’s score) and OCT-derived microvascular parameters among different ischemic time groups, one-way ANOVA or nonparametric test followed by Tukey’s post hoc test was used. Correlations between OCT-derived vascular parameters and histological indices were assessed using nonparametric Spearman’s rank correlation analysis. Receiver operating characteristic (ROC) curve analysis was performed to evaluate the ability of OCT-derived vascular parameters to predict severe intestinal mucosal injury (defined as ${\mathrm{Chiu}}^{\prime }s\;\mathrm{score}\geq 3$).^7^^,^^24^ This threshold was selected to distinguish early or mild mucosal injury from histologically evident structural mucosal damage characterized by marked epithelial lifting and villous injury, consistent with the established Chiu scoring system. The area under the ROC curve (AUC) and 95% confidence intervals (CI) were calculated. The optimal cutoff value was determined using the Youden index. A two-tailed *P* value <0.05 was considered statistically significant.

## 3 Results

### 3.1 Monitoring Changes in Intestinal Mucosal Microcirculation Using OCTA during Acute Intestinal Ischemia in Mice

OCT imaging enabled clear visualization of the intestinal mucosal microvascular network *in vivo*. Representative OCT angiography images revealed well-organized vascular structures under baseline conditions. Following mesenteric artery ligation, local blood circulation ceases due to erythrocyte stasis. Consequently, a significant reduction in visible vessel count is observed as ischemia duration increases. Although blood flow does not completely halt in some vessels, the significantly reduced erythrocyte flow velocity results in insufficient signal variation between consecutive B-scans to generate distinct flow signals. These vessels merely present as a dim background with low signal-to-noise ratio. As shown in Fig. 2, blood flow signals in normal intestinal tissue are bright and well-defined. With the progression of ischemia, vessels of different diameters disappear sequentially. At 4 h post-mesenteric artery ligation, OCTA images reveal a uniformly dark background in the intestinal wall, where microvessels and small vessels are no longer detectable, leaving only sporadic isolated large vessels. These findings demonstrate that repeated OCT imaging at different time points was feasible in the same intestinal segment, with consistent image quality and no apparent gross motion artifacts after frame rejection and *en face* median filtering, although no image-based registration was applied (see limitations), supporting the suitability of OCT for dynamic monitoring of intestinal microcirculation during acute ischemia.

### 3.2 Changes in the Length and Perfused Vessel Density of Intestinal Mucosal Vessels with Different Diameters during Acute Intestinal Ischemia in Mice

Given that blood vessels of different diameters perform distinct physiological functions within tissues, a systematic analysis of the sequence in which these vessels are lost during ischemia may provide new insights into the mechanisms underlying ischemic injury of the intestinal tissue. Therefore, we quantified the temporal changes in vessel length and perfused vessel density of vessels with different diameters during ischemia [Fig. 3(a)]. Figure 2 shows the original *en face* OCTA angiograms, in which each pixel value represents the decorrelation amplitude of the flow signal, whereas Fig. 3(a) shows the skeletonized vessel maps obtained after vessel segmentation, in which each pixel is color-coded by the local vessel diameter (see color bar). The two presentations are therefore not directly comparable in terms of pixel intensity. The results showed a significant reduction in both the length and VPD of intestinal mucosal microvessels with prolonged ischemia time (Tables 1 and 2). Furthermore, we specifically investigated the differences in the trends of length change among vessels of different diameters over time. The results revealed that at all measured ischemic time points, the degree of reduction in both length and perfusion density was significantly greater for vessels with diameters>50  μm compared with those with diameters<20  μm (P<0.05) [Figs. 3(b)–3(e)]. Therefore, these findings suggest that intestinal mucosal vessels are heterogeneously affected, with larger-caliber vessels exhibiting greater vulnerability to ischemic conditions.

**Fig. 2:**
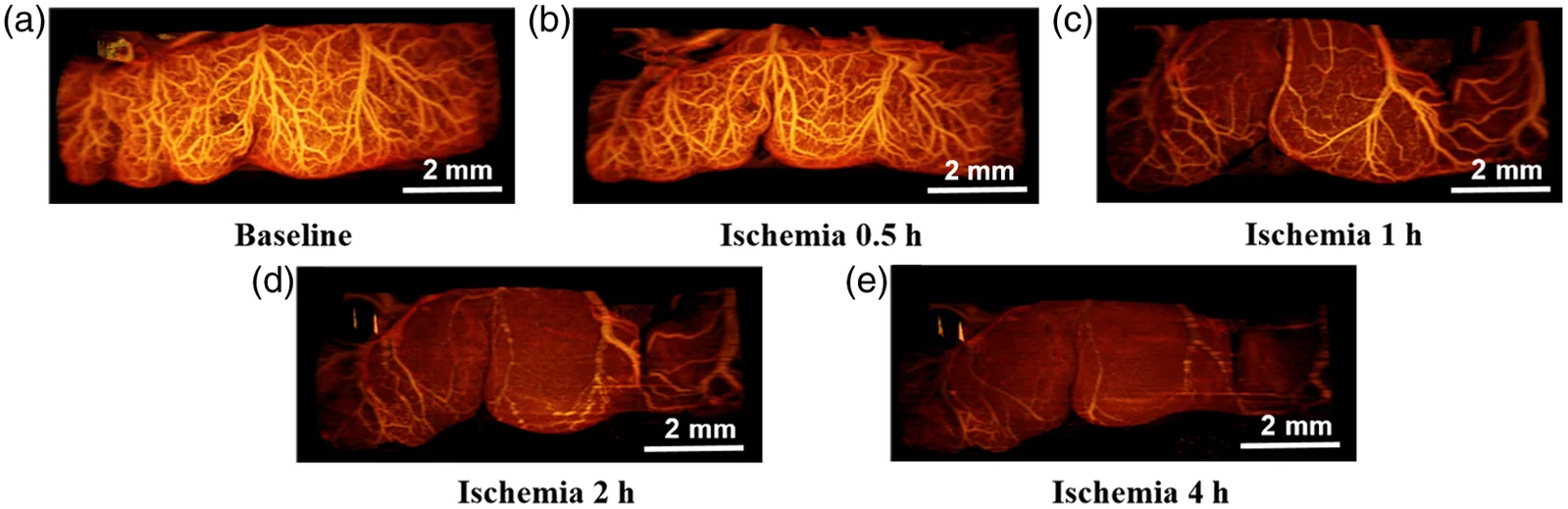
OCTA images showing temporal changes in intestinal mucosal microcirculation in mice with acute mesenteric ischemia. (a) Baseline. (b) Ischemia 0.5 h. (c) Ischemia 1 h. (d) Ischemia 2 h. (e) Ischemia 4 h. Scale bars are shown in each panel; the field of view of each *en face* angiogram is 10  mm(horizontal) x5  mm(vertical).

**Fig. 3:**
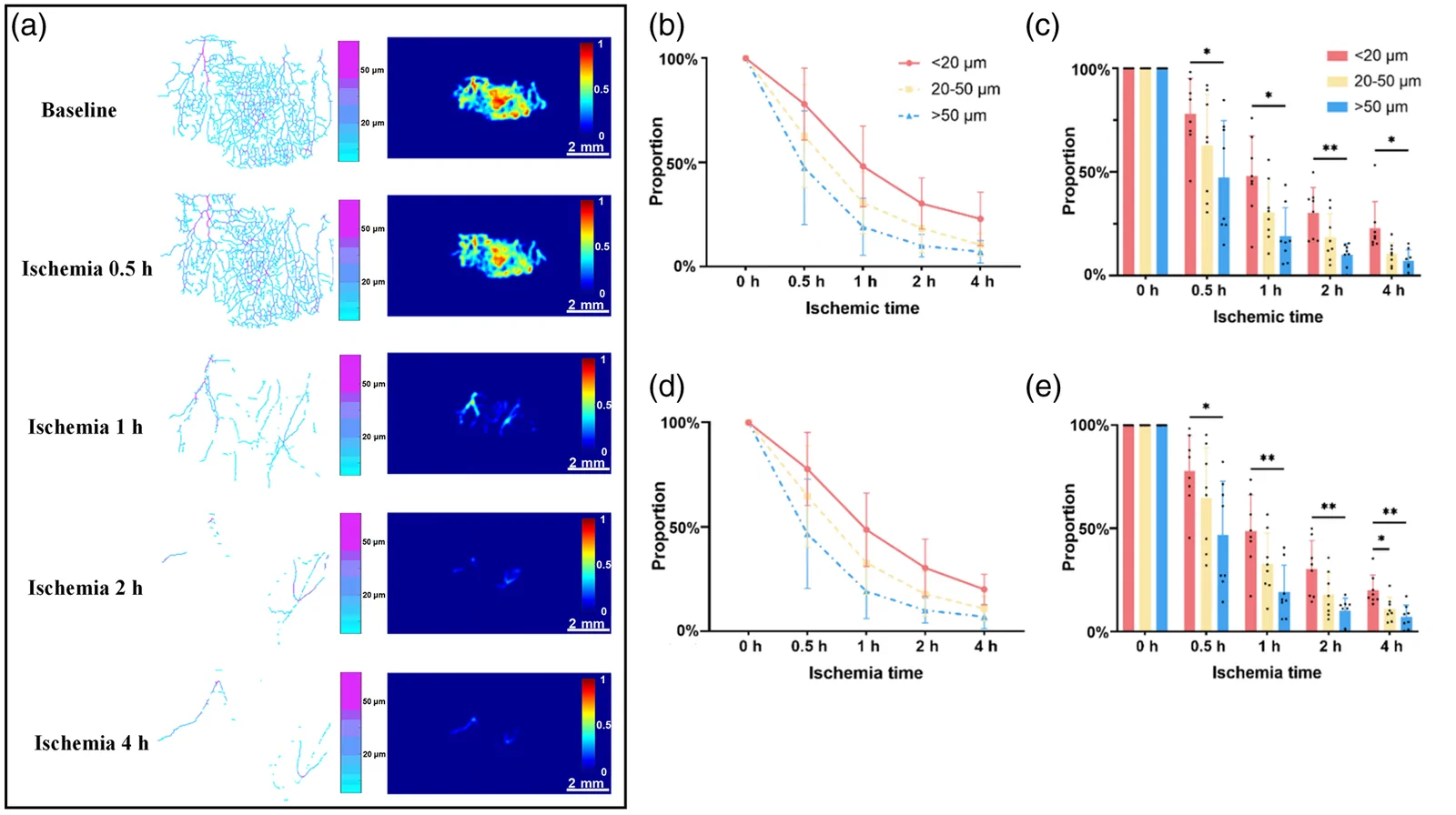
OCTA demonstrates temporal changes in intestinal mucosal microvascular length and VPD in mice with acute mesenteric ischemia. (a) Representative OCTA images showing dynamic changes in intestinal mucosal microvascular length and VPD at different ischemic time points. (b)–(c) Changes in intestinal mucosal microvascular length across different vessel diameter categories over time following ischemia. (d)–(e) Changes in intestinal mucosal perfused vessel density (VPD) across different vessel diameter categories over time following ischemia. * P<0.05, ** P<0.01. In panel (a), vessels are displayed as skeleton maps in which the color encodes the local vessel diameter (<20 um, 20 to 50 um and >50 um, as indicated by the color bar); the value of each pixel in panel (a) therefore represents vessel diameter rather than flow signal and should not be interpreted in the same way as the angiograms in Fig. 2. Scale bars are shown in panel (a).

**Table 1:** Absolute vessel length of intestinal mucosal microvessels across different vessel diameter categories over time following ischemia (unit, mm).

Microvascular diameter	Baseline	Ischemia 0.5 h	Ischemia 1 h	Ischemia 2 h	Ischemia 4 h
<20 μm	63.63±7.41	50.68±8.19 ^a^	33.82±7.80 ^a^	20.65±4.18 ^a^	13.52±2.58 ^a^
20 to 50 μm	90.98±6.87	61.71±8.83 ^a^	31.79±6.21 ^a^	17.12±4.31 ^a^	10.26±2.07 ^a^
>50 μm	92.84±16.90	52.61±14.49 ^a^	19.75±5.96 ^a^	9.65±2.52 ^a^	5.79±1.38 ^a^

a P<0.05 versus the baseline group.

**Table 2:** Absolute perfused vessel density (VPD) of intestinal mucosal microvessels across different vessel diameter categories over time following ischemia (unit, %).

Microvascular diameter	Baseline	Ischemia 0.5 h	Ischemia 1 h	Ischemia 2 h	Ischemia 4 h
<20 μm	5.01±0.62	3.92±0.62 ^a^	2.54±0.56 ^a^	1.60±0.36 ^a^	1.04±0.22 ^a^
20 to 50 μm	14.31±1.37	9.61±1.59 ^a^	4.97±1.07 ^a^	2.74±0.76 ^a^	1.60±0.36 ^a^
>50 μm	31.19±6.34	17.59±4.98 ^a^	6.76±2.17 ^a^	3.20±0.78 ^a^	2.08±0.53 ^a^

a P<0.05 versus the baseline group.

### 3.3 OCT-Derived Vascular Alterations Precede Histological Injury

To further investigate the sequence of microvascular changes and pathological injury of intestinal mucosa, mice were divided into five groups: sham control, 0.5-h ischemia, 1-h ischemia, 2-h ischemia, and 4-h ischemia, followed by assessment of the aforementioned vascular and histological indices. HE staining results showed that compared to the sham group, intestinal mucosal villus height was significantly reduced in the 1-h ischemia group (533.64±12.81 versus 654.53±8.11, P<0.0001), the 2-h ischemia group (392.27±12.84 versus 654.53±8.11, P<0.0001), and the 4-h ischemia group (174.83±10.84 versus 654.53±8.11, P<0.0001) [Fig. 4(a)]. The crypt depth in the mice of the 4-h ischemia group (179.35±4.69 versus 219.26±4.55, P<0.0001) was significantly lower than that in the sham group mice [Fig. 4(b)]. Histopathological evaluation demonstrated a progressive increase in mucosal injury with prolonged ischemia. The Chiu’s score remained low at early ischemic time points (ischemia 0.5 and 1 h, P>0.05) and increased significantly at later stages (ischemia 2 and 4 h, P<0.05), indicating the development of overt intestinal mucosal damage over time [Fig. 4(c)]. OCT images showed that <20  μm diameter intestinal mucosal vessels length in the mice of the 2-h ischemia group (43.01±5.02 versus 118.64±8.48, P<0.01) and the 4-h ischemia group (29.04±3.35 versus 118.64±8.48, P<0.0001) was significantly lower than that in the sham group. Compared with the sham group mice, the 20 to 50  μm (154.80±13.53 versus 245.30±9.87, P<0.0001) and >50  μm (98.05±13.34 versus 173.30±12.61, P<0.0001) diameter intestinal mucosal vessels length significantly decreased in the mice of the 0.5-h ischemia group, which was consistent in the 1-h, 2-h, and 4-h ischemia groups [Fig. S1(A) in Supplementary Material]. We further examined the changes in perfusion density of vessels with different diameters. Compared with the sham group mice, the VPD of <20  μm diameter intestinal mucosal vessels significantly decreased in the mice of the 2-h (1.83±0.22% versus 5.09±0.36%, P=0.002) and 4-h (1.05±0.13% versus 5.09±0.36%, P<0.0001) ischemia group. A significant decrease in the VPD of 20 to 50  μm (10.16±2.52% versus 15.18±2.35%, P<0.0001) and >50  μm (19.51±2.95% versus 32.70±3.72%, P<0.0001) diameter intestinal mucosal vessels was found in the 0.5-h ischemia group mice compared with the sham group. Consistent with this, the same reduction was observed in the 1-h, 2-h, and 4-h ischemia groups [Fig. S1(B) in Supplementary Material]. Both vessel length and VPD of 20 to 50  μm and >50  μm mucosal vessels showed a significant reduction before a noticeable increase in the Chiu’s score was observed. Furthermore, Figs. 4(d)–4(i) reveal a clear dissociation between microvascular alterations and histological injury. Specifically, significant reductions in vessel length and VPD of medium- and large-diameter vessels (20 to 50  μm and >50  μm) were detected at ischemic stages when the Chiu’s score had not yet increased significantly, whereas small-diameter vessels (<20  μm) exhibited relatively delayed changes. These findings indicate that OCT-derived microvascular alterations precede the development of histologically detectable mucosal injury during acute mesenteric ischemia.

**Fig. 4:**
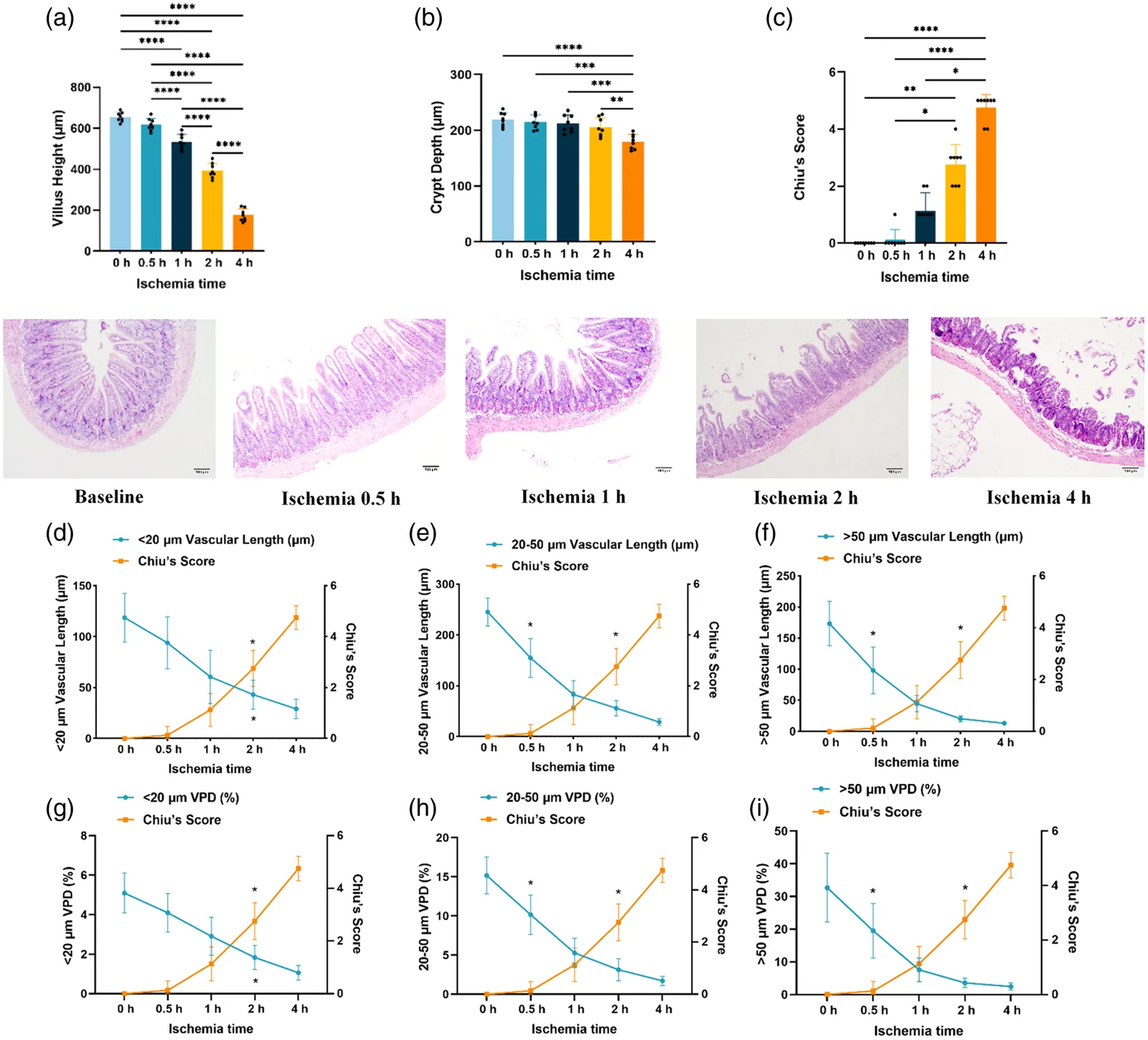
Comparison of histological and microvascular parameters among different groups. Differences in intestinal mucosal villus height (a), crypt depth (b), and Chiu’s score (c), among groups. (d)–(i) Line graphs showing changes in intestinal mucosal microvascular length and VPD across different vessel diameter categories, along with alterations in intestinal mucosal Chiu’s score. * P<0.05, ** P<0.01, *** P<0.001, **** P<0.0001.

### 3.4 Correlations between Microvascular Parameters and Intestinal Mucosal Injury

To further examine the relationship between microcirculatory alterations and intestinal mucosal injury, correlation analyses were performed between OCT-derived vascular parameters and histological indices. Significant positive correlations were observed between the lengths and perfused vessel density (VPD) of intestinal mucosal vessels and both villus height and crypt depth (P<0.05). These correlations were consistently present across all three vessel diameter categories (<20  μm, 20 to 50  μm, and >50  μm) (Fig. 5). Conversely, the lengths and VPD of intestinal mucosal vessels exhibited significant negative correlations with the Chiu’s score (P<0.05), indicating that greater microvascular impairment was associated with more severe histological injury (Fig. 5). Together, these findings demonstrate a close quantitative association between OCT-derived microcirculatory parameters and the severity of intestinal mucosal damage.

**Fig. 5:**
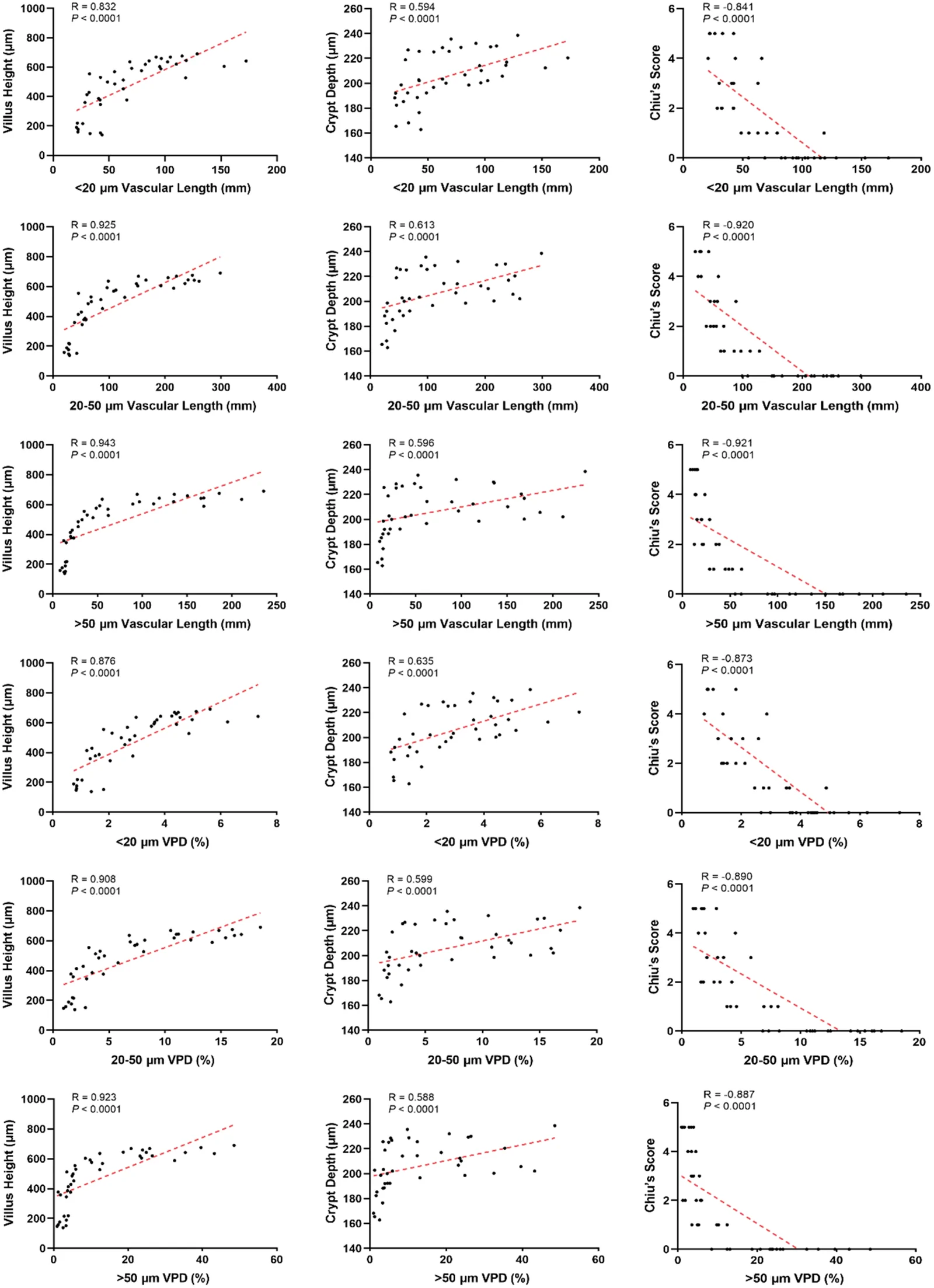
Correlation analysis between microvascular parameters and intestinal mucosal histological indices.

### 3.5 Diagnostic Performance of OCT-Derived Vascular Parameters for Identifying Severe Mucosal Injury

ROC curve analysis demonstrated that OCT-derived vascular parameters had significant diagnostic performance for severe mucosal injury (${\mathrm{Chiu}}^{\prime }s\;\mathrm{score}\geq 3$). Among all vessel diameter categories, vessel length of >50  μm vessels showed the highest diagnostic performance, with an AUC of 0.956 (95% CI: 0.890 to 1.000). The optimal cutoff value was 30.65 [Fig. 6(c)]. However, small-diameter (<20  μm) and medium-diameter (20 to 50  μm) vessels showed lower AUC values [Figs. 6(a) and 6(b)]. Similarly, VPD of >50  μm vessels yielded the highest AUC of 0.927 (95% CI: 0.840 to 1.000). The optimal cutoff value was 4.55 [Fig. 6(f)]. Medium-diameter vessels (20 to 50  μm) demonstrated moderate diagnostic ability, whereas small-diameter vessels (<20  μm) showed lower AUC values [Figs. 6(d) and 6(e)]. Notably, the optimal cutoff identified an early perfusion threshold below which the probability of structural mucosal destruction increased markedly.

**Fig. 6:**
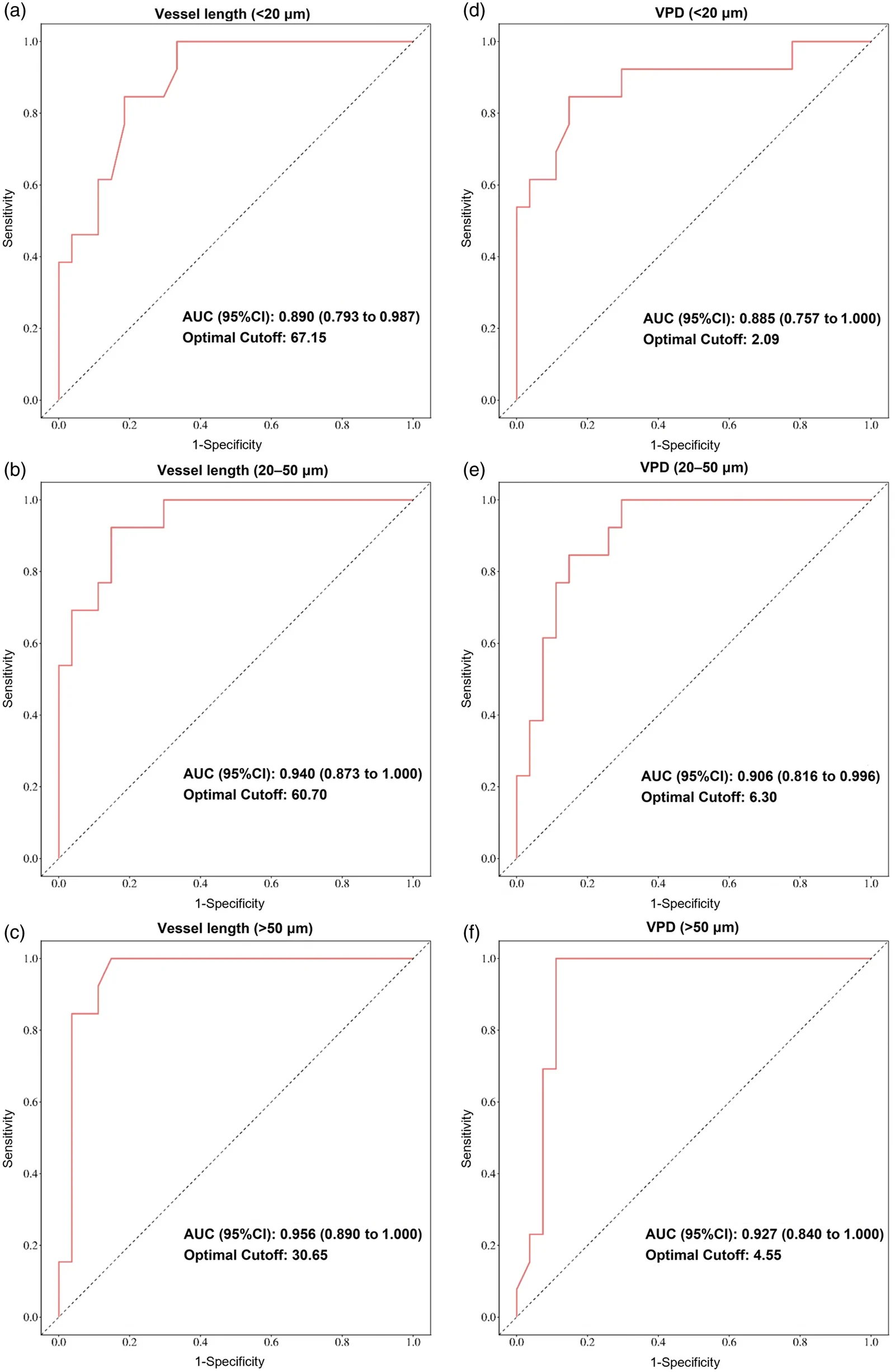
Receiver operating characteristic (ROC) curves of OCTA-derived microvascular parameters for discriminating severe intestinal mucosal injury (${\mathrm{Chiu}}^{\prime }s\;\mathrm{score}\geq 3$). (a)–(c) ROC curves for vessel length in vessels with diameters of <20  μm, 20 to 50  μm, and >50  μm, respectively. (d)–(f) ROC curves for perfused vessel density (VPD) in vessels with diameters of <20  μm, 20 to 50  μm, and >50  μm, respectively.

## 4 Discussion and Conclusion

In this study, we systematically evaluated the value of optical coherence tomography (OCT) for dynamic monitoring of intestinal mucosal microcirculation in a mouse model of AMI. We demonstrated that ischemia-induced microvascular impairment was both time-dependent and vessel diameter–dependent. Specifically, vessels with diameters>50  μm exhibited a more rapid and pronounced reduction in vessel length and VPD than smaller vessels (<20  μm). Histological analysis revealed a progressive aggravation of mucosal injury with prolonged ischemia, characterized by a gradual reduction in villus height and crypt depth and an increase in the Chiu’s score. Importantly, decreases in vascular parameters of 20 to 50  μm and >50  μm vessels preceded the elevation of the Chiu’s score, indicating that microvascular dysfunction occurs earlier than overt histological injury. Moreover, vascular length and VPD across all vessel diameter categories were positively correlated with villus height and crypt depth and negatively correlated with the Chiu’s score, highlighting a close association between microcirculatory integrity, mucosal structural preservation, and the severity of ischemic injury. Both vessel length and VPD of >50  μm mucosal vessels showed the best performance for discriminating severe mucosal injury (${\mathrm{Chiu}}^{\prime }s\;\mathrm{score}\geq 3$). Through these integrated methods, we clarified the role of OCT in detecting early microcirculatory dysfunction and to provide experimental evidence supporting the potential use of OCTA for intraoperative assessment of intestinal viability.

We found that vessels >50  μm exhibited a significantly faster decline in length and VPD than vessels <20  μm as ischemia progressed. This diameter-dependent pattern of microvascular injury is closely related to the hierarchical organization of intestinal mucosal blood supply. Large-diameter vessels (>50  μm), which mainly include mucosal arteriolar branches and villous arterial trunks, are responsible for delivering oxygen and nutrients to the entire villus. Although these vessels have relatively thick walls, they are highly sensitive to ischemia because they are directly affected by blood flow interruption and vasospasm following mesenteric artery ligation.^25^ By contrast, small-diameter vessels (<20  μm), which primarily consist of capillary networks at the villus tip and around crypts, possess a greater compensatory capacity during early ischemia and may temporarily maintain structural integrity through residual perfusion and diffusion-based oxygen supply.^26^ Consequently, their length and VPD decline more slowly. This pattern of “large-vessel first injury followed by small-vessel involvement” is consistent with the known pathophysiological progression of acute intestinal ischemia.^27^^,^^28^

A notable finding of this study is the temporal dissociation between microvascular alterations and histologically detectable mucosal injury. Significant reductions in vascular parameters of 20 to 50  μm and >50  μm vessels were observed before Chiu’s score increased. This supports the concept that microvascular dysfunction represents an initiating event in ischemic injury. The Chiu’s score, a widely used histopathological grading system for intestinal ischemic injury, primarily reflects established morphological damage such as villous edema, epithelial shedding, and crypt destruction.^7^ Structural disintegration of epithelial cells requires a critical threshold of ATP depletion, cytoskeletal breakdown, and membrane disruption.^29^^,^^30^ By contrast, microcirculatory dysfunction represents an initial event in ischemic injury.^31^ These results indicate that OCT enables detection of early microvascular impairment before severe mucosal damage becomes apparent on conventional histology.

Correlation analyses further demonstrated significant positive associations between OCT-derived vascular parameters and villus height and crypt depth, as well as significant negative correlations with Chiu’s score. These findings quantitatively confirm the close coupling between microcirculatory integrity and mucosal structural preservation. Villi are the primary sites of nutrient absorption and rely on continuous oxygen and nutrient delivery from the microvasculature, whereas crypts contain intestinal stem cells responsible for epithelial renewal and repair.^32^^,^^33^ Progressive microvascular impairment therefore leads to insufficient metabolic support, resulting in villous atrophy and crypt damage, which is reflected by increasing histological injury scores.^34^^,^^35^

Among the different vessel diameter categories, large-diameter vessels (>50  μm) showed the fastest decline and the strongest correlation with villus height, suggesting that these vessels may serve as particularly sensitive indicators of mucosal injury severity. Although significant correlations were also observed for small-diameter vessels, their correlation coefficients were lower likely because capillary network damage represents a later manifestation of microcirculatory failure. Medium- and large-diameter vessels, as upstream components of the mucosal microvascular network, appear to be more responsive to early ischemic insults and more closely associated with overall mucosal injury.^36^^,^^37^

The ROC analysis further demonstrated the potential discriminatory value of OCTA-derived vascular parameters for severe mucosal injury. The high AUC values observed for vessel length and VPD in vessels >50  μm suggest that alterations in larger-caliber perfused vessels are closely associated with the development of substantial mucosal structural damage. Unlike conventional histological assessment, which detects established structural damage, OCT-derived perfusion parameters provide a quantitative threshold that may facilitate early risk stratification. This discriminatory ability supports the potential role of OCTA in identifying the severity of intestinal mucosal injury and provides a rationale for its future evaluation as an adjunctive intraoperative tool for intestinal viability assessment. The translational relevance of our findings is better considered in the context of intraoperative assessment of intestinal viability rather than detection of AMI at the moment of disease onset. By the time patients with AMI undergo surgical exploration, mesenteric arterial occlusion and varying degrees of intestinal injury may already be established. The critical surgical challenge is therefore to determine the extent of irreversible injury while preserving as much viable intestine as possible. Conventional assessment based on bowel color, peristalsis, mesenteric pulsation, and bleeding remains subjective, particularly in segments with equivocal viability. A realistic implementation strategy would involve the use of a compact handheld or laparoscopic-compatible contact OCT/OCTA probe applied directly to the exposed bowel surface during surgical exploration. Such a configuration would allow rapid, localized imaging of suspicious bowel segments and adjacent areas while avoiding the need for contrast agents. This concept is supported by previous clinical work showing the feasibility of intraoperative multimodal OCT from the serosal surface in patients with acute mesenteric ischemia and demonstrating differences among nonischemic, viable ischemic, and necrotic bowel segments.^14^

The penetration depth of OCT represents an important technical limitation for clinical translation. The imaging depth of our system is ∼2  mm, whereas the thickness of the human bowel wall varies substantially and may increase further in the presence of edema, inflammation, hemorrhage, or distension. Therefore, OCT should not currently be considered a technique capable of comprehensively visualizing the entire bowel wall in every patient. Rather, its potential value lies in high-resolution assessment of superficial and mid-wall microstructure and perfusion, particularly from the serosal surface, where alterations in mucosal and intramural vascular perfusion may provide indirect information regarding tissue viability. Previous gastrointestinal OCT studies have demonstrated that OCT can visualize multiple layers of the bowel wall, although imaging depth remains a recognized limitation.^38^^–^^40^ Future development of longer-wavelength, higher-sensitivity systems, together with optimized focusing and probe design, may improve penetration depth and reduce attenuation in edematous bowel.

Our findings demonstrate that OCTA-derived microvascular parameters are closely associated with the progression and severity of intestinal ischemic injury, providing a potential imaging basis for intraoperative viability assessment. Although the present study does not establish an OCT-guided resection protocol, these findings provide a rationale for future studies investigating whether OCT/OCTA can delineate the transition between viable and severely ischemic bowel and thereby assist intraoperative determination of bowel resection margins.

Several limitations should be acknowledged. First, this study was conducted in a murine model, and extrapolation to human AMI requires further validation. Second, we focused on vascular length and VPD, whereas other functional parameters such as blood flow velocity and vascular permeability were not assessed. Future studies integrating multimodal functional imaging may provide a more comprehensive evaluation of intestinal microcirculation. Third, a time-matched longitudinal sham group was not included in the first part of our study. Although a separate sham group was included in the second part to provide a nonischemic reference for OCT/OCTA and histological measurements, it was not followed longitudinally under the same prolonged anesthesia and repeated imaging conditions as the first-part animals. Future studies incorporating a time-matched longitudinal sham group with identical anesthesia, surgical exposure, and imaging procedures will be important to further isolate ischemia-specific microvascular changes. Fourth, no image-based registration or bulk-motion correction was applied to the OCTA data. This was a deliberate choice rather than an oversight. Contact imaging under maintained anesthesia already suppressed the majority of bulk motion, so the benefit of registration was limited; registering every B-scan would have been computationally expensive and time-consuming; and aggressive registration risks attenuating the decorrelation signal that carries the flow information, thereby reducing angiographic contrast. Motion was instead addressed by discarding the least-correlated of five repeated frames at each position and by median filtering the *en face* angiograms. Residual respiratory, cardiac, and peristaltic motion may nevertheless persist and attenuate the decorrelation signal of low-flow vessels, biasing both vessel length and VPD toward underestimation. The inter-frame interval of 6.25 ms makes the decorrelation contrast sensitive to slow flow but also susceptible to such residual motion. Because any residual bias would act in the same direction at all ischemic time points and in all diameter categories, it is unlikely to account for the temporal trend or the diameter-dependent ordering reported here. Finally, although the second part of our study provides information on microvascular parameters during the early post-reperfusion period and may not reflect sustained microvascular recovery. Future studies incorporating serial post-reperfusion imaging and independent assessment of intestinal viability are warranted to determine the potential utility of OCT/OCTA for intraoperative decision-making regarding bowel preservation and resection.

In conclusion, OCT enables dynamic and quantitative assessment of intestinal mucosal microvascular alterations during acute mesenteric ischemia. Microvascular impairment occurs in a diameter-dependent manner and precedes overt histological mucosal injury, with large-caliber vessels showing the most pronounced alterations. OCT-derived vascular parameters are also closely associated with histological injury severity and demonstrate good discriminatory performance for severe mucosal injury. These findings support the potential role of OCT as an adjunctive intraoperative tool for assessing intestinal viability and provide a foundation for future studies aimed at identifying ischemic transition zones and optimizing bowel resection boundaries.

## Supplementary Material

10.1117/1.JBO.31.11.116001.s01

## Data Availability

The original contributions presented in the study are included in the article; further inquiries can be directed to the corresponding author.
