# Supplementary material for: OCT angiography characterizes time- and diameter-dependent intestinal microvascular alterations in acute mesenteric ischemia
Source: J Biomed Opt. 2026 Nov 9;31(11):116001. doi: 10.1117/1.JBO.31.11.116001 (PMC13746245; doi:10.1117/1.JBO.31.11.116001)
Supplement: Supplementary file 1 [file JBO_031_116001_SD001.docx]

**OCT Angiography Characterizes Time- and Diameter-Dependent Intestinal Microvascular Alterations in Acute Mesenteric Ischemia**

**Yu Tian^1,†^, Feifan Wang^2,†^, Jian Liu^3,†^, Nan Li^4^, Mingshuo Zhang^1,5^, Rui Ding^1,6^, Yimin Wang^2,*^**

^1^ Department of Hand & Foot Surgery, First Hospital of Qinhuangdao, Qinhuangdao 066000, China.

^2^ Department of General Surgery, First Hospital of Qinhuangdao, Qinhuangdao 066000, China.

^3^ School of Control Engineering, Northeastern University at Qinhuangdao, Qinhuangdao 066000, China.

^4^ Department of Ophthalmology, Qinhuangdao Maternal and Child Health Hospital, Qinhuangdao 066000, China.

^5^ Hebei North University, Zhangjiakou 075000, China.

^6^ Hebei Medical University, Shijiazhuang 050017, China.

**^*^** Correspondence: Yimin Wang, drwangyimin@126.com

**^†^** These authors contributed equally to this work.

Figure S1 Differences in intestinal mucosal microvascular length (A) and VPD (B) across different vessel diameter categories among groups.
